# The combination of phase angle and age has a good diagnostic value for sarcopenia in continuous ambulatory peritoneal dialysis patients

**DOI:** 10.3389/fnut.2022.1036796

**Published:** 2022-11-15

**Authors:** Ye Chen, Jinlan Wu, Lei Ran, Dan Yu, Xi Chen, Maodong Liu

**Affiliations:** ^1^Department of Nephrology, The Third Hospital of Hebei Medical University, Shijiazhuang, China; ^2^Department of Nutrition, The Third Hospital of Hebei Medical University, Shijiazhuang, China

**Keywords:** continuous ambulatory peritoneal dialysis (CAPD), the body composition analysis, phase angle, sarcopenia, diagnostic value, age

## Abstract

**Aims:**

There are limited studies on phase angle and sarcopenia in continuous ambulatory peritoneal dialysis patients. So, we want to explore the association between phase angle and sarcopenia and find a more sensitive indicator for diagnosing sarcopenia.

**Methods:**

We included 101 continuous ambulatory peritoneal dialysis patients from March 2022 to August 2022 and measured the phase angle and body composition by bioelectrical impedance analysis. All patients had their handgrip strength measured. Then, we divided patients into the sarcopenia (*n* = 30) group and non-sarcopenia (*n* = 71) group according to the sarcopenia diagnostic strategy formulated by the Asian Working Group for Sarcopenia. We used logistic regression to explore the risk factors of sarcopenia. We applied Receiver-operating characteristics curves to determine the diagnostic accuracy of these risk factors.

**Results:**

After adjustments for sex, age, diabetes, BMI, extracellular water ratio, extra water, serum creatinine, total kt/v, and residual kt/v, phase angle correlated to handgrip strength and lowered limb muscle mass but not to skeletal muscle mass, upper arm muscle circumference, upper limb muscle mass and appendicular skeletal muscle mass index. In the multivariate logistic model, low phase angle and older age are risk factors for sarcopenia. The AUROC of phase angle for sarcopenia is 0.79 (95%CI, 0.70–0.86, *P* < 0.01) for both sexes, 0.70 and 0.85 for females and males. After we combined age and phase angle as diagnostic indicators of sarcopenia, the AUROC is 0.91 (95%CI, 0.83–0.96, *P* < 0.0001) in both sexes, 0.89 and 0.93 for females and males.

**Conclusion:**

This study illustrates that age 52 or older is an independent risk factor for sarcopenia in continuous ambulatory peritoneal dialysis patients. Phase angle can act as a predictor of sarcopenia in those patients. But the combination of age and phase angle is more valuable in diagnosing sarcopenia.

## Introduction

Sarcopenia is the loss of muscle mass, strength, or muscle function, which was initially thought to be an age-related disease (1). In contrast, recent studies showed that some inflammatory diseases, such as tumors and organ failure, could increase the risk of sarcopenia (2). In addition, sarcopenia is one of the most important complications in patients undergoing peritoneal dialysis (PD) due to a micro-inflammatory state, inadequate caloric intake, uremic toxin accumulation, volume overload, and PD process (3), with a prevalence of 4–15.5% (4, 5). Several studies demonstrated that sarcopenia was associated with physical disabilities, higher mortality, cardiovascular events, and chronic kidney disease (CKD) falls. In 2015, a prospective analysis of hemodialysis (HD) patients presented a higher risk of disabilities in sarcopenia than the non-sarcopenia group (6). Kamijo et al. (7) enrolled 119 PD patients and found that sarcopenia was associated with a higher risk of mortality (survival mortality rate per 500 days: 0.667 vs. 0.971 between sarcopenia and non-sarcopenia group, *p* < 0.001). Then, a study of elderly HD patients showed that sarcopenia patients presented a higher risk of falls than non-sarcopenia patients. According to a prospective observational study (8), sarcopenia was a strong predictor of death (HR, 6.99; 95% CI, 1.84–26.58; *p* = 0.004) and cardiovascular events (HR, 4.33; 95% CI, 1.51–12.43; *p* = 0.006). In addition, a multicenter analysis confirmed a correlation between sarcopenia and a high risk of hospitalization in HD patients (RR, 2.07, 95%CI, 1.48–2.88, *P*< 0.001). In summary, using easily accessible and cheaper instruments to test the nutritional status of patients is extremely important for diagnosing and preventing sarcopenia early in clinical practice.

Bioelectrical impedance analysis (BIA) can easily and quickly measure body composition and distinguish muscle, fat, and water (9). Thus, it has been widely used for body composition measurement. BIA is more valuable in CKD due to the volume overload in those patients. Recent guidelines recommend that BIA be used to estimate muscle mass in diagnosing sarcopenia (2). Phase angle (PhA) was an indicator measured by BIA and has been gaining attention because it reflected the cellular function and the distribution of intracellular and extracellular water (10). Several studies found it correlated to muscle strength and function (11, 12). Thus, the current evidence encouraged research on using PhA in nutrition care and diagnosis, particularly in sarcopenia. Many studies have explored the relationship between sarcopenia and PhA in different diseases, including PD patients. However, there are few studies on Chinese continuous ambulatory peritoneal dialysis (CAPD) patients. After all, the phase angle value varies from one race to another (13).

In clinical practice, there are many CAPD patients in our nephrology clinic. Exploring easy ways to find sarcopenia in these patients draws our attention. This study wants to explore the association between PhA and sarcopenia in Chinese CAPD patients. Meanwhile, we hope to explore the diagnostic value of sarcopenia and find an optimal cut-off in these patients.

## Materials and methods

### Study design and patient recruitment

This cross-sectional study was carried out between March 2022 and August 2022. It evaluated CAPD patients in the PD Clinic of The Third Hospital of Hebei Medical University, Shijiazhuang, Hebei Province. Inclusion criteria were patients aged > 18 years, on regular dialysis > 3 months, and with regular follow-up. Patients with acute infection at any site (respiratory, digestive, urinary), acute cardiovascular disease, malignancy, active liver disease, tuberculosis, AIDS, syphilis, other systemic diseases, surgery, trauma, or disability resulting in insufficient grip strength and body composition analysis data were excluded. The Ethics Committee approved the study protocol of The Third Hospital of Hebei Medical University (number: 20210931). All subjects signed an informed consent form.

### Anthropometric measurements

The Clinical nutrition test analyzer (AINST 2018, Taizhou city, Jiangsu province, China) records the patient’s body weight with a precision of 1 g (we will subtract the weight of peritoneal fluid when measuring). Height was measured by a Portable size measuring instrument with an accuracy of 0.1 cm.

### Body composition analysis and phase angle

We use a clinical nutrition test analyzer to determine body composition and PhA based on the BIA method. Subjects were asked to fast for at least 8 h and empty their urine and stool in the early morning of the test. Measurement method: Clean the electrode contact surface of the instrument with an alcoholic cotton ball, and measure the height and weight of the subject; the subject is barefoot, wipe the soles of both feet and palms of both hands with an alcohol cotton ball, make both feet and hands contact the silver electrode contact surface of the instrument to the maximum extent, and introduce the current. Start to measure and read total water, extra water (OH), protein, skeletal muscle mass (SMM), body fat, appendicular skeletal muscle mass index (ASMI), extracellular water ratio (E/T), PhA, and other data.

### Baseline variables

We obtained sociodemographic backgrounds, including name, sex, and age through face-to-face interviews. Clinical data, including dialysis vintage, blood pressure, and year diagnosed with end-stage renal disease (ESRD), come from electronic or paper cases from our center. Routine laboratory tests, including high-sensitive C-reactive protein (Hs-CRP), serum hemoglobin, and so on, were collected from medical records. Blood samples are collected on an empty stomach on the morning of the day of the body composition measurement.

### Grip strength

Handgrip strength (HGS) is measured by a Grip Strength meter (CAMRY, MODEL: EH101). We ask subjects to turn their palms inward with the dial facing outward. Their bodies stand upright, and their arms hang down naturally. The grip meter should not contact their bodies and clothes. Then, use the force of the muscle group of the leading hand to measure three times and take the average value.

### Total kt/V_*urea*_ assessment

A 24-h urine sample and dialysis fluid specimen were collected, and fasting venous blood was taken on the morning of the following day. Kt/V urea is calculated. At the same time, professionals checked and recorded the Dialysis protocol, urine volume, and ultrafiltration volume for the day.


$$\mathrm{Total}\,\frac{K}{V}=\mathrm{Dialysis}=\frac{\mathrm{Kt}}{V}+\mathrm{Residual}\,\mathrm{renal}\,\frac{\mathrm{Kt}}{V}$$

$$\mathrm{Dialysis}\,\frac{\mathrm{Kt}}{V}=\frac{\mathrm{dialysate}\,\mathrm{urea}\,\left(\mathrm{mmol}/L\right)}{\mathrm{serum}\,\mathrm{urea}\,\left(\mathrm{mmol}/L\right)}\times 24\,h\,\mathrm{abdominal}\,\mathrm{dialysate}\,\mathrm{excretion}\,\mathrm{volume}\,\left(L\right)\times 7/\mathrm{body}\mathrm{weight}\left(\mathrm{kg}\right)\times 0.60\left(\mathrm{male}\right)/\;0.55\,\left(\mathrm{female}\right)$$

$$\mathrm{Residual}\,\mathrm{renal}\,\frac{\mathrm{Kt}}{V}=\frac{24\,h\,\mathrm{urine}\,\mathrm{urea}\,\left(\mathrm{mmol}/L\right)}{\mathrm{serum}\,\mathrm{urea}\,\left(\mathrm{mmol}/L\right)}\times 24\,h\,\mathrm{abdominal}\,\mathrm{dialysate}\,\mathrm{excretion}\,\mathrm{volume}\,\left(L\right)\times 7/\mathrm{body}\mathrm{weight}\left(\mathrm{kg}\right)\times 0.60\left(\mathrm{male}\right)/\;0.55\,\left(\mathrm{female}\right)$$

### Sarcopenia diagnosis

According to the Asian sarcopenia diagnostic strategy formulated by the Asian Working Group for Sarcopenia (AWGS), the subjects should meet the following two criteria of the BIA test:1. ASMI = appendicular skeletal muscle (kg)/height (m^2^): Males < 7.0 kg/m^2^, Females < 5.7 kg/m^2^; 2. Handgrip strength: Males < 26 kg, females < 18 kg.

### Statistical analysis

We analyzed the data using the statistical software IBM SPSS Statistics version 26.0 (SPSS Inc, Chicago, IL, United States). Continuous variables with normal distribution were presented as mean ± standard deviation (SD), whereas skewed data were presented as median (q1–q3). The number of cases (percentage) was used for categorical counts.

The Pearson product-moment correlation assesses the correlation between PhA and diagnostic indicators of sarcopenia. Logistic regression analysis is performed to determine the independent predictors of sarcopenia. A multivariate analysis is adjusted for age, total Kt/V, residual kidney Kt/V, basal metabolic, OH, E/T, and serum creatinine.

We use a receiver-operating characteristics (ROC) curve analysis to determine diagnostic accuracy for PhA, age, and the two combined to detect sarcopenia. The area under the curve (AUC) indicates the discriminative power of the test. The Youden index calculated the best cut-off value in the AUC. *P* < 0.05 was considered a statistically significant difference.

## Results

### The basic information about continuous ambulatory peritoneal dialysis patients

We enrolled 101 patients on CAPD who came to our outpatient clinic from March 2022 to August 2022. including 53 males (52.48%) and 48 females (47.52%), with a mean age of 51.31 ± 12.68 years, and 30 patients were diagnosed with sarcopenia (Table 1).

**TABLE 1:** Comparison of variables in sarcopenia and non-sarcopenia abdominal dialysis patients.

Variables	Total	Sarcopenia group (*n* = 30)	Non-sarcopenia group (*n* = 71)	t/z/χ^2^ value	*P*-value
**Baseline variables**					
Age	51.31 ± 12.68	62.93 ± 9.10	46.39 ± 10.63	7.44	**< 0.01**
Sex	Male 53 (52.48%)	Male 13 (43.33%)	Male 40 (56.34%)	1.430^a^	0.23
DM	Yes 20 (19.80%)	Yes 9 (30%)	Yes 11 (15.49%)	2.795^a^	0.09
SBP	140 (130–150)	139.97 ± 12.92	140 (130–150)	-0.21	0.83
DBP	90 (86–97.5)	90 (86–92.25)	90 (86–99)	-0.83	0.40
Dialysis age (month)	30.0 (14.5–69)	21.5 (15–71)	35 (14.5–65.5)	-0.23	0.82
**Bioelectrical impedance analysis**					
BFP (%)	25.53 ± 9.47	27.5 ± 11.04	24.69 ± 8.67	1.368	0.17
WHR	0.82 ± 0.009	0.82 ± 0.11	0.82 ± 0.09	0.182	0.85
AC (cm)	28.8 ± 3.32	28.01 ± 2.98	29.13 ± 3.41	-1.57	0.12
AMC (cm)	23.47 ± 2.45	22.75 ± 2.06	23.84 (21.68-25.83)	-1.51	0.13
BMI (kg/m^2^)	23.63 ± 3.97	23.46 ± 3.14	23.72 ± 4.3	-0.326	0.75
HGS (kg)	25.90 ± 8.98	16.69 ± 4.41	29.79 ± 7.44	10.97	**< 0.001**
BMR (kcal)	1372.18 (1219.84–1591.2)	1328.35 ± 169.56	1456.74 (1232.42–1615.41)	-2.09	**0.04**
PhA (°)	5.4 (4.6–5.8)	4.62 ± 0.81	5.5 (5.3–6)	-4.58	**< 0.01**
Cell mass (kg)	29.32 (24.81–35.43)	27.84 ± 4.96	32.11 (25.34–37.05)	-2.32	**0.02**
LBM (kg)	46.4 (39.35–56.54)	44.37 ± 7.85	50.31 (39.93–57.66)	-2.09	**0.04**
Protein (kg)	8.85 (7.49–10.69)	8.4 ± 1.5	9.69 (7.65–11.18)	-2.32	**0.02**
SMM (kg)	24.69 (20.59–30.26)	23.35 ± 4.52	27.24 (21.08–31.74)	-2.32	**0.02**
Muscle mass (kg)	43.46 (37.02–53.4)	41.7 ± 7.49	47.28 (37.53–54.52)	-2.15	**0.03**
RaLM (kg)	2.33 (1.76–2.95)	2.16 ± 0.53	2.48 (1.79–3.04)	-2.278	**0.02**
LaLM (kg)	2.19 (1.79–2.92)	2.14 ± 0.51	2.4 (1.81–2.97)	-1.89	0.06
RlLM (kg)	7.72 ± 2.3	6.65 (5.56–8.08)	8 ± 2.37	-2.01	**0.045**
LlLM (kg)	7.38 (5.81–9.26)	6.71 (5.49–8.2)	7.97 ± 2.32	-2.02	**0.04**
ASMI	4.65 ± 0.84	4.38 ± 0.64	4.77 ± 0.90	2.46	**0.02**
OH (L)	1.21 (0.77–1.82)	1.43 (1.21–1.93)	1.09 (0.71–1.73)	-2.73	**< 0.01**
E/T	0.4 (0.39–0.41)	0.4 (0.4–0.42)	0.39 (0.39–0.4)	-4.3	**< 0.01**
**Routine laboratory tests**					
Total Kt/V	1.78 ± 0.29	1.90 ± 0.34	1.73 ± 0.25	2.74	**< 0.01**
Residual kidney kt/V	0.106 (0–0.47)	0.28 (0–0.82)	0 (0–0.36)	-2.71	**< 0.01**
Dialysis kt/V	1.5 ± 0.40	1.48 ± 0.42	1.51 ± 0.4	-0.35	0.73
Hb (g/L)	110.0 (102.5–120)	111 (104–118.7)	110 (101–122)	-0.39	0.69
Hs-CRP (mg/L)	3.2 (1.7–9.82)	3.2 (1.78–7.51)	3.24 (1.67–10.16)	-0.20	0.84
ALB (g/L)	38.53 ± 4.28	36.90 (33.97–39.62)	39.48 ± 0.44	-3.2	**< 0.01**
Na^+^(mmol/L)	140.66 ± 3.02	139.16 ± 3.24	141.29 ± 2.71	3.397	**0.001**
P^+^(mmol/L)	1.77 ± 0.46	1.53 (1.36–1.90)	1.81 ± 0.44	-1.99	**0.047**
Cr (μmol/L)	1020.37 ± 289.87	819.15 ± 216.3	1105.4 ± 275.62	-5.06	**< 0.01**
BUN (mmol/L)	20.62 ± 4.89	20.20 ± 4.69	20.79 ± 4.99	-0.55	0.58
HCO_3_**^–^**(mmol/L)	25.19 ± 2.76	24.73 ± 2.53	25.88 (23.41–27.18)	-1.252	0.214
PA (mmol/L)	365 ± 70.74	332.79 ± 82.87	378.61 ± 60.59	-3.1	**< 0.01**
TG (mmol/L)	1.45 (1.12–2.17)	1.39 (1.12–2.5)	1.5 (1.11–2.17)	-0.25	0.80
TC (mmol/L)	5.01 ± 1.22	5.07 ± 1.38	4.98 ± 1.15	0.31	0.76
HDL (mmol/L)	1.24 (1.01–1.51)	1.34 ± 0.39	1.17 (1.01–1.45)	-1.0	0.32
LDL (mmol/L)	2.79 ± 0.76	2.85 ± 0.73	2.77 ± 0.78	0.5	0.62
PTH (pg/mL)	417.9 (211.75–691.25)	333.4 (171.025–530.8)	536.27 ± 344.51	-2.03	0.042

DM, Diabetic Mellitus; SBP, Systolic blood pressure; DBP, Diastolic blood pressure; BFP, Body fat percentage; WHR, Waist-Hip ratio; AC, Upper arm circumference; AMC, Upper arm muscle circumference; BMR, Basal metabolic rate; PhA, Phase angle; LBM, Lean body mass; SMM, Skeletal muscle mass; RaLM, right upper extremity muscle mass; LaLM, left upper extremity muscle mass; RlLM, right lower extremity muscle mass; LlLM, left lower extremity muscle mass; ASMI, extremity skeletal muscle mass index; OH, Extra water; E/T, Extracellular water ratio; Hb, Hemoglobin; Hs-CRP, Hypersensitive C-reactive protein; ALB, Serum albumin; Na^+^, Serum sodium; P^+^, Serum phosphorus; Cr, Serum creatinine; BUN, Serum urea; HCO**_3_^–^**, Bicarbonate; PA, Prealbumin; TG, Triglycerides; TC, Total cholesterol; HDL, High-density lipoprotein; LDL, Low-density lipoprotein; PTH, Parathyroid hormone. The bold values refer to *P*-values of less than 0.05, indicating statistically significant differences.

#### The comparison between the sarcopenia group and the non-sarcopenia group

Age is significantly higher in the sarcopenia group than in the non-sarcopenia group. Some other demographic feathers, such as gender, diabetes, blood pressure, and dialysis age, did not differ significantly between the two groups. The body composition analysis shows that basal metabolic rate, PhA, cell mass, de-fatted body weight, protein, skeletal muscle, and muscle mass are all lower in the sarcopenia than in the non-sarcopenia group. The total Kt/V and residual kidney Kt/V are higher in the sarcopenia group than in the non-sarcopenia group. Laboratory test shows that serum calcium, serum sodium, serum albumin, serum pre-albumin, serum creatinine, and parathyroid hormone (PTH) are all lower in the sarcopenia group than in the non-sarcopenia group; In contrast, excess water and E/T are higher in the sarcopenia group than in the non-sarcopenia group (Table 1).

#### The relationship between PhA and diagnostic indicators of sarcopenia

**FIGURE 1:**
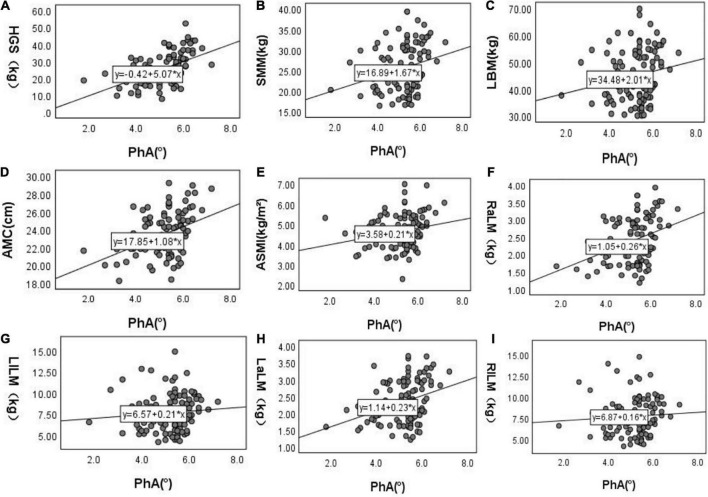
Scatter plots of PhA and related variables.

After doing the scatter plot (Figure 1), Pearson correlation analysis shows a positive correlation between PhA with HGS, SMM, ACM, ASMI, and upper limb muscle mass but not with muscle mass and lower limb muscle mass (Model 1). However, after adjusting for age, diabetes, BMI, E/T, OH, serum creatinine level, total Kt/V, and residual Kt/V, PhA is positively correlated to HGS and lower limb muscle mass but not with ASMI, muscle mass, upper limb muscle mass, SMM and AMC (Model 7, Table 2).

**TABLE 2:** The Pearson correlation between PhA with Indicators related to sarcopenia.

	Model													
Variables	Model 1		Model 2		Model3		Model 4		Model 5		Model 6		Model 7	
	*r*	*p*	*r*	*p*	*r*	*p*	*r*	*p*	*r*	*p*	*r*	*p*	*r*	*p*
PhA × HGS	0.52	<0.001	0.28	<0.01	0.23	0.024	0.20	0.047	0.18	0.08	0.25	0.015	0.24	**0.02**
PhA × ASMI	0.23	0.023	-0.126	0.22	-0.23	0.029	-0.21	0.041	-0.02	0.07	-0.18	0.09	-0.16	0.12
PhA × muscle mass	0.19	0.054	-0.15	0.14	0.26	0.01	0.22	0.035	0.19	0.07	0.17	0.1	0.08	0.43
PhA × RaLM	0.36	<0.001	0.22	0.03	0.3	0.004	0.26	0.01	0.24	0.02	0.22	0.03	0.14	0.17
PhA × LaLM	0.30	<0.01	0.16	0.11	0.26	0.01	0.21	0.04	0.07	0.19	0.19	0.08	0.1	0.34
PhA × RlLM	0.07	0.52	-0.29	0.004	0.361	<0.001	0.4	<0.001	0.37	<0.001	0.35	0.001	0.28	**0.007**
PhA × LlLM	0.08	0.39	-0.26	0.01	0.39	<0.001	0.43	<0.001	0.41	<0.001	0.38	<0.001	0.32	**0.002**
PhA × SMM	0.26	0.008	-0.043	0.68	0.26	0.01	0.22	0.035	0.19	0.07	0.17	0.1	0.08	0.43
PhA × AMC	0.40	<0.001	0.49	<0.001	0.26	0.01	0.24	0.02	0.22	0.04	0.22	0.04	0.15	0.15

Model 1: Univariate correlation; Model 2: adjusted for sex, age, diabetes, and BMI; Model 3: adjusted for sex, age, diabetes, BMI, and Extracellular water ratio; Model 4: adjusted for sex, age, diabetes, BMI, Extracellular water ratio and extra water; Model 5: adjusted for sex, age, diabetes, BMI, Extracellular water ratio, extra water, and serum creatinine; Model 6: adjusted for sex, age, diabetes, BMI, Extracellular water ratio, extra water, serum creatinine, and total kt/v; Model 7: adjusted for sex, age, diabetes, BMI, Extracellular water ratio, extra water, serum creatinine, total kt/v, and residual kt/v. The bold values refer to *P*-values of less than 0.05, indicating statistically significant differences.

#### Multivariate logistic regression between PhA and sarcopenia

**FIGURE 2:**
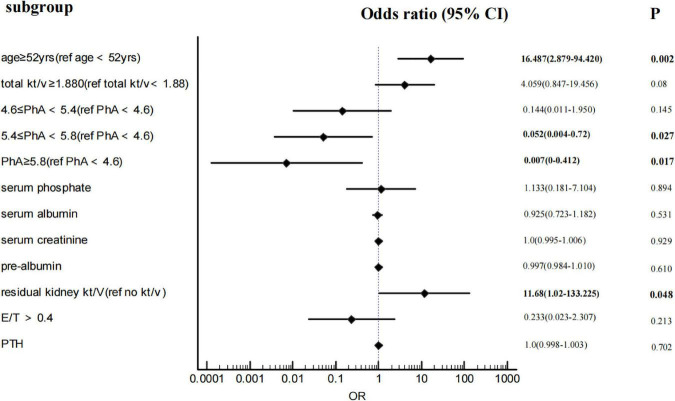
Forest plot of binary logistic regression results.

After adjustments for age, total Kt/V, residual kidney Kt/V, serum phosphorus, OH, E/T, and serum creatinine, albumin, and pre-albumin, multivariate binary logistic regression (Figure 2) shows that with PhA<4.6 as the baseline level, the ORs of 4.6 ≤ PhA<5.4, 5.4 ≤ PhA<5.8, and PhA ≥ 5.8 are 0.144 (95%CI 0.011–1.95), 0.052 (95%CI 0.004–0.72), and 0.007 (95%CI 0–0.412). The *P*-value is less than 0.05, except for the 5.4 ≤ PhA<5.8. In addition, age ≥ 52years and residual kidney kt/v>0 are independent risk factors for sarcopenia. As the diagnostic criteria included HGS and ASMI, HGS and muscle-related indicators such as SMM and muscle mass were excluded from the logistic regression.

### Receiver-operating characteristics curve analysis

**FIGURE 3:**
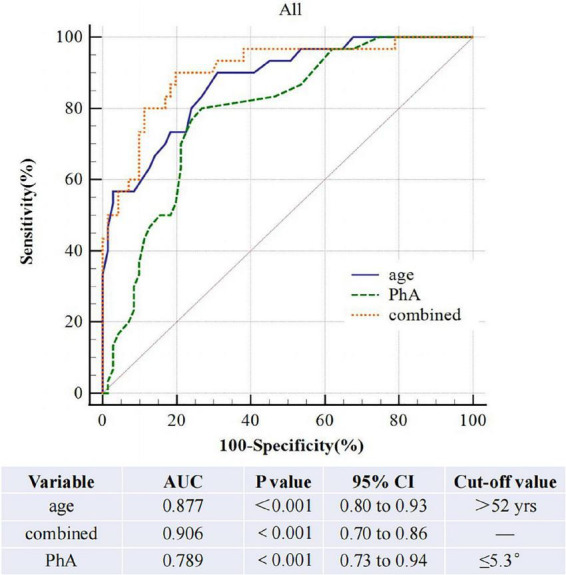
Roc curve comparsion of three diagnostic indicators in all people.

**FIGURE 4:**
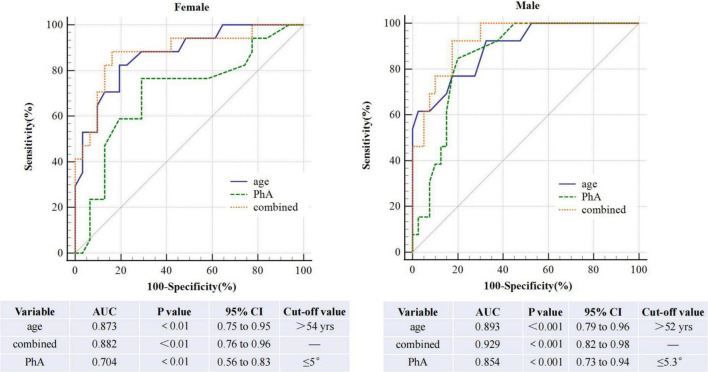
Roc curve comparison of three diagnostic indicators in females and males.

The AUROC of PhA for sarcopenia is 0.79 (95% CI, 0.70–0.86, *P*<0.01, Figure 3) in both sexes, 0.70 and 0.85 for women and men. The optimal cut-off value is ≤ 5.3°(sensitivity 80%, specificity 73.24%) in all people, 5 °and 5.3° for women and men (Figure 4). On the other hand, the AUROC of age is 0.88 (95%CI, 0.8–0.93, *P* < 0.001) in both sexes, 0.87 and 0.89 for women and men; the cut-off point of age for sarcopenia is > 52 years (sensitivity 90%, specificity 69%) in all, 54 and 52 in female and male. Finally, we used age and phase angle as a joint diagnostic indicator for sarcopenia, with an AUROC of 0.91 (95%CI, 0.83–0.96, *P* < 0.0001) in both sexes, 0.88 and 0.93 for females and males. And the difference in AUROC between PhA and PhA-age is statistically significant in all (*p* = 0.002, Table 3) and women, but not statistically significant in men.

**TABLE 3:** Comparison of the AUROC of the three diagnostic indicators for all patients.

Compare variables	Difference between areas	95%CI	*P*
PhA∼combined	0.12	0.04–0.19	**<0.01**
age∼combined	0.03	-0.01–0.07	0.14
PhA∼age	0.09	-0.02– 0.19	0.09

The bold values refer to *P*-values of less than 0.05, indicating statistically significant differences.

## Discussion

Primary sarcopenia is an age-related syndrome characterized by reduced SMM, muscle strength, and muscle function (1), which is associated with various adverse outcomes such as falls, disabilities, fractures, and death. Some studies (14, 15) illustrated that worsened kidney function is associated with increased sarcopenia, especially in dialysis patients. Thus, early diagnosis and intervention of sarcopenia are essential for dialysis patients. The BIA has been used for the last decades for body composition analysis of its simplicity and less harm to the human body (16). PhA, one of the parameters of BIA, reflects the number of cells and cells’ integrity and has been shown to correlate with nutritional status and sarcopenia.

The main finding of our study is that compared with the non-sarcopenia group, sarcopenia patients have a lower PhA. Meanwhile, in multivariate analysis, PhA is associated with sarcopenia. The result is consistent with some previous studies. In 2012 (17), Marini et al. enrolled 207 elderly and compared the PhA between non-sarcopenia and sarcopenia groups identified by dual-energy X-ray absorptiometry (DXA). They also found that PhA was lower in the sarcopenia group, but PhA showed a positive association with ASMI, which is different from our result. After adjusting for age, diabetes, BMI, E/T, OH, serum creatinine level, total Kt/V, and residual Kt/V, we find PhA is correlated to HGS and lower limb muscle mass but not with ASMI. This may be due to differences in the way ASMI was measured. Yamada et al. (13) demonstrated a moderate correlation between PhA with HGS and sarcopenia. Do et al. revealed that PhA negatively correlated to sarcopenia after a multivariate logistic regression in kidney transplant patients. Research on 200 patients with PD (18) demonstrated that PhA is independently associated with muscle mass, strength, and sarcopenia. These data suggest that PhA is a valuable indicator for sarcopenia, including our results. However, there are some mixed findings. Pessoa et al. illustrated that PhA was unaffected by sarcopenia, SMI, HGS, and walk speed in older women (19). Another study in kidney transplant patients (20) also thought PhA measured by BIA was not associated with sarcopenia but with HGS. The relationship between PhA and sarcopenia may exist only in a particular disease.

The ROC curve analysis shows that the AUROC is 0.854 and the cut-off value of PhA in sarcopenia is 5.3° in all, 0.854 and 5.3°in men, and 0.704 and 5° in women. Similar studies have been done before. Yamada et al. illustrated that the average PhA for sarcopenia is 4.05°for men and 3.55°for women in community-de-welling Japanese older adults (13). A study (21) on elderly inpatients showed the optimal PhA cut-off value was ≤ 4.55° (sensitivity 70% and specificity 65.9%). In 2019 (22), a value ≤ 5.05°was indicated in cirrhosis (sensitivity 71.3% and specificity 65.5%). Kosoku et al. demonstrated a value of ≤ 4.46 in kidney transplant patients, with a sensitivity of 74% and specificity of 70% (23). In these studies, men’s cut-off values were higher than women’s, consistent with our findings. Recently, a survey about PD showed the AUROC of PhA for sarcopenia was 0.73 (95% CI, 0.67–0.79, *p* < 0.001). The optimal cut-off value was identified as ≤ 4.4°(sensitivity 81.3%, specificity 59.6%) (18), which was lower than our result. This may be due to the total average age of our central dialysis patients (51.31 ± 12.68 years) being lower than that of the patients included in the study (55.5 ± 12.2 years). Besides this, race can also influence the PhA. Regrettably, they did not discuss men and women separately. This is the first study to examine the diagnostic value of phase angle for sarcopenia individually for men and women in CAPD patients. More extensive clinical studies are needed.

Age is an essential risk factor for sarcopenia in CAPD patients. ROC curve analysis shows age is a great predictor, and the cut-off value is ≥ 52years in all patients. We again discuss men and women separately (Figure 4). The AUROC in males is 0.893, and the cut-off point is ≥ 52 years; meanwhile, in females is 0.873, and the cut-off point is ≥ 54 years. The cut-off value is higher for women than for men. This may be due to men losing more muscle area per year than women (-4.9 ± 4.7% vs. -3.4 ± 7.9%) (24). But in another research, they demonstrated no difference in muscle loss between men and women older than 70 years after 5 years of follow-up (25). The cut-off age for sarcopenia is lower in men, which warrants further investigation.

Most studies in the past focused on sarcopenia in the elderly 60 years or older. Compared With primary sarcopenia, chronic kidney disease patients are more susceptible to sarcopenia due to the presence of protein degradation, dietary restriction, reduced activity level, and other elements (26). Muscle loss occurs younger in HD patients compared with the average population (10). Ozkayar et al. also administrated sarcopenia that occurred early in renal transplant recipients (27). Therefore, we should pay more attention to younger CAPD patients.

Except for age, male gender (15), lower BMI (20), diabetes, longer dialysis duration (28), malnutrition (such as lower serum albumin/pre-albumin) (7, 29), and high-sensitive C-reactive protein (Hs-CRP) (30, 31) were found associated with sarcopenia also in a multivariate logistic regression. Consistent with previous studies, serum albumin and pre-albumin are lower in sarcopenia than in the non-sarcopenia group, but not sex, BMI, dialysis duration, diabetes, and Hs-CRP. The multivariate regression analysis shows that they are not associated with sarcopenia. This may be caused by the small sample size of our study and the different diagnostic criteria. After adjustments for other components, the prevalence of sarcopenia is 11 times higher in patients with residual kidney Kt/V > 0 than in those without residual kidney Kt/V (OR = 11.68, 95% CI:1.02–133.225, *P* = 0.048). To our knowledge, this is the first research finding residual kidney function as a risk factor for sarcopenia in CAPD patients. Clinicians require that the protein intake of CAPD patients with residual renal function is lower than those without residual renal function to protect residual kidney function. Therefore, we speculate that the increased risk of sarcopenia in patients with residual renal function may be related to protein intake. But our study did not include dietary surveys of patients, which is a shortcoming of the study—looking forward to more in-depth research on residual kidney function, dietary intake, and sarcopenia in the future.

Meanwhile, serum creatinine is lower in the sarcopenia group than in the non-sarcopenia group. These are different from our conjectures. However, binary logistic regression results showed no correlation between serum creatinine and sarcopenia.

To find a better diagnostic tool, we used an age-PhA combined indicator to make ROC curves for sarcopenia and compared the three curves. Then, we find that the integrated indicator has the largest AUROC. This suggests that the age-PhA combined indicator has a more excellent diagnostic value than PhA in both men and women. Ishii et al. first combined age, grip strength, and calf circumference and demonstrated it had a good diagnostic value in older adults; the AUROC of this tool is 0.939 for men and 0.909 for women (32). Much focus has been placed on the diagnostic value of PhA for sarcopenia in different diseases, including PD patients. Our study is the first research to combine age and phase angle as diagnostic indicators of sarcopenia in CAPD patients. Therefore, more research is needed to confirm.

The advantages of our study are the following: 1. Several previous studies on sarcopenia had used incomplete criteria, but they used muscle mass alone. Our study uses two variables (HGS and SMI) to diagnose sarcopenia. 2. We use consensus and definitions of sarcopenia for Asia populations. 3. For the first time, we combine phase angle and age and found an excellent diagnostic value. 4. We discuss men and women separately.

Our study also has some limitations: 1. This is a single-center study with a small sample size, and our results still need to be confirmed by a large multicenter trial. 2. BIA is used instead of DXA for the body composition analysis of DXA. A guideline suggests that performing would be more reasonable than using a BIA to measure muscle mass in CAPD patients. 3. BIA is contraindicated in patients with permanent pacemakers or other implanted electronic devices. Its accuracy cannot be demonstrated in patients with amputations, and cerebrovascular disease, the generalizability of our findings to these specific populations is limited.

## Conclusion

In conclusion, older age, residual kidney kt/v, and lower phase angle are risk factors for sarcopenia. PhA can be used as a predictor of muscle mass and strength in CAPD patients; we also developed a more convenient and accurate way to diagnose sarcopenia in CAPD patients.

## Data availability statement

The original contributions presented in the study are included in the article/Supplementary material, further inquiries can be directed to the corresponding author.

## Ethics statement

The studies involving human participants were reviewed and approved by the Ethics Committee of the Third Hospital of Hebei Medical University. The patients/participants provided their written informed consent to participate in this study.

## Author contributions

YC collected body composition analysis data from patients, performed statistical analysis of the data, and wrote the first draft. JW was in charge of screening the patients. ML and DY were responsible for the experimental design and revision of the manuscript. LR was responsible for collecting data on patients’ grip strength and following up with the patients. XC collected and checked all the data. All authors contributed to the article and approved the submitted version.

## Supplementary material

Supplementary Data Sheet 1 The original data.
